# Medical Classes

**Published:** 1844-02

**Authors:** 


### MEDICAL CLASSES.

In our last number, we gave the reputed size of the classes at several of the medical schools in the west. From our contemporaries beyond the mountains, we learn, that the Atlantic Schools are fuller than usual. The lecture term recently closed in the Geneva Medical College with a Class of from 150 to 180. A writer in the Boston Medical and Surgical Journal reports about 500 students at the two schools in New York city, and 600 in the two Philadelphia schools. This statement is confirmed by the editor of the Medical Examiner, who says, "at the two schools in Philadelphia the number of students is greater than at any former season. The University of Pennsylvania has more than her average class, and the number at the Jefferson Medical College exceeds that of last year, by nearly one hundred." Y.

